# (5-Amino­isophthalato-κ*N*)triaqua­(1,10-phenanthroline-κ^2^ *N*,*N*′)cobalt(II) trihydrate

**DOI:** 10.1107/S1600536810040560

**Published:** 2010-10-20

**Authors:** Kou-Lin Zhang, Guo-Wang Diao, Seik Weng Ng

**Affiliations:** a College of Chemistry and Chemical Engineering, Yangzhou University, Yangzhou 225002, People’s Republic of China; b Department of Chemistry, University of Malaya, 50603 Kuala Lumpur, Malaysia

## Abstract

The Co^II^ atom in the title compound, [Co(C_8_H_5_NO_4_)(C_12_H_8_N_2_)(H_2_O)_3_]·3H_2_O, is six-coordinated in a CoN_3_O_3_ octa­hedral geometry; the water-coordinated Co^II^ atom is chelated by the *N*-heterocycle. An inter­molecular N—H⋯O hydrogen bond occurs. The carboxyl­ate entity coordinates through the amino group. The carboxyl­ate donor unit, coordinated and uncoordinated water mol­ecules inter­act through O—H⋯O and N—H⋯O hydrogen bonds, generating a tightly-held three-dimensional cage-like network.

## Related literature

For related structures, see: He *et al.* (2006 ▶); Wu *et al.* (2002*a* ▶,*b* ▶).
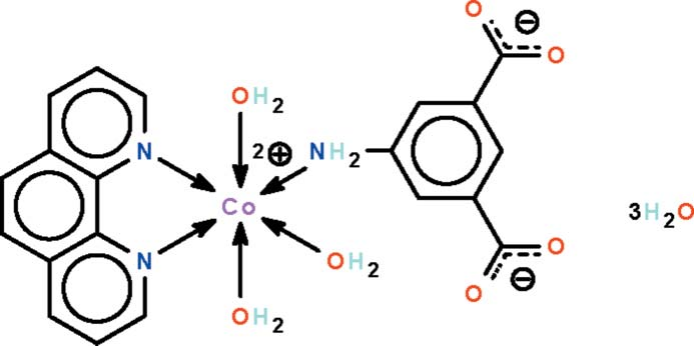


## Experimental

### 

#### Crystal data

[Co(C_8_H_5_NO_4_)(C_12_H_8_N_2_)(H_2_O)_3_]·3H_2_O
*M* *_r_* = 526.36
Monoclinic, 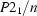

*a* = 10.1182 (2) Å
*b* = 13.9659 (2) Å
*c* = 16.2850 (2) Å
β = 95.827 (1)°
*V* = 2289.34 (8) Å^3^
*Z* = 4
Mo *K*α radiation
μ = 0.81 mm^−1^
*T* = 293 K
0.24 × 0.22 × 0.18 mm

#### Data collection

Bruker APEXII area-detector diffractometer
Absorption correction: multi-scan (*SADABS*; Sheldrick, 1996 ▶) *T*_min_ = 0.803, *T*_max_ = 1.000
18954 measured reflections
5683 independent reflections
5120 reflections with *I* > 2σ(*I*)
*R*_int_ = 0.027

#### Refinement

*R*[*F* ^2^ > 2σ(*F* ^2^)] = 0.028
*wR*(*F* ^2^) = 0.086
*S* = 1.04
5683 reflections
363 parameters
14 restraints
H atoms treated by a mixture of independent and constrained refinement
Δρ_max_ = 0.47 e Å^−3^
Δρ_min_ = −0.46 e Å^−3^

### 

Data collection: *APEX2* (Bruker, 2004 ▶); cell refinement: *SAINT* (Bruker, 2004 ▶); data reduction: *SAINT*; program(s) used to solve structure: *SHELXS97* (Sheldrick, 2008 ▶); program(s) used to refine structure: *SHELXL97* (Sheldrick, 2008 ▶); molecular graphics: *X-SEED* (Barbour, 2001 ▶); software used to prepare material for publication: *publCIF* (Westrip, 2010 ▶).

## Supplementary Material

Crystal structure: contains datablocks global, I. DOI: 10.1107/S1600536810040560/si2299sup1.cif

Structure factors: contains datablocks I. DOI: 10.1107/S1600536810040560/si2299Isup2.hkl

Additional supplementary materials:  crystallographic information; 3D view; checkCIF report

## Figures and Tables

**Table 1:** Hydrogen-bond geometry (Å, °)

*D*—H⋯*A*	*D*—H	H⋯*A*	*D*⋯*A*	*D*—H⋯*A*
O1w—H1w1⋯O6w^i^	0.83 (1)	1.90 (1)	2.734 (1)	175 (2)
O1w—H1w2⋯O2^ii^	0.84 (1)	1.81 (1)	2.646 (1)	173 (2)
O2w—H2w1⋯O5w^i^	0.83 (1)	1.93 (1)	2.761 (1)	174 (2)
O2w—H2w2⋯O4^iii^	0.85 (1)	1.94 (1)	2.790 (1)	173 (2)
O3w—H3w1⋯O5w^iv^	0.84 (1)	2.16 (2)	2.909 (2)	148 (2)
O3w—H3w2⋯O3^ii^	0.85 (1)	1.86 (1)	2.694 (1)	167 (2)
O4w—H4w1⋯O6w^v^	0.85 (1)	1.98 (1)	2.811 (2)	169 (2)
O4w—H4w2⋯O2^iv^	0.85 (1)	2.05 (1)	2.864 (2)	161 (3)
O5w—H5w1⋯O1	0.85 (1)	1.89 (1)	2.716 (2)	164 (2)
O5w—H5w2⋯O3^vi^	0.85 (1)	1.90 (1)	2.719 (1)	161 (2)
O6w—H6w1⋯O1	0.85 (1)	1.82 (1)	2.665 (1)	174 (2)
O6w—H6w2⋯O4^iii^	0.85 (1)	1.94 (1)	2.784 (1)	174 (2)
N1—H1*N*1⋯O4w	0.85 (1)	2.06 (1)	2.906 (2)	169 (2)
N1—H1*N*2⋯O4^iii^	0.84 (1)	2.30 (1)	3.110 (2)	161 (2)

Symmetry codes: (i) 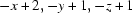; (ii) 
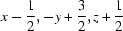
; (iii) 
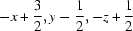
; (iv) 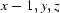; (v) 
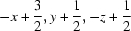
; (vi) 
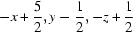
.

**Fig. 1.**
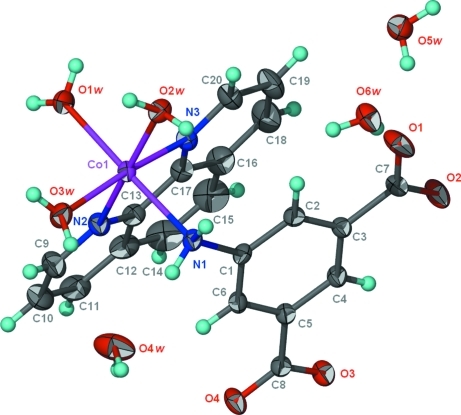
Thermal displacement ellipsoid plot of (I) at the 70% probability level; hydrogen atoms are shown as spheres of arbitrary radii.

**Fig. 2.**
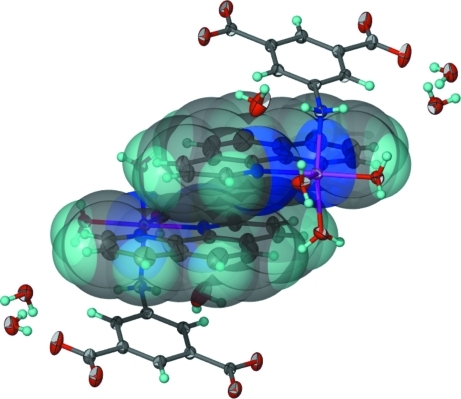
Two formula units of (I) showing π···π interactions about a center-of-inversion.

## supplementary crystallographic information

### Comment

The dianion of 5-aminoisophthalic acid binds to cobalt(II) in a bridging *µ*_4_-manner in the monoaqua derivative (Wu *et al.*, 2002*a*), and the carboxyl oxygen as well as the amino nitrogen atoms are all involved in bonding in the three-dimensional network structure. A diaqua dihydrate has also been reported; the compound has the monoanion in *µ*_2_ bridging that gives rise to a chain motif (Wu *et al.*, 2002*b*). The 4,4'-bipyridine spacer ligand lowers the dimensionality of cobalt 5-aminoisophthalate, and the diqua adduct, which crystallizes as a DMF solvate, exists as linear chains (He *et al.*, 2006). The present 1,10-phenanthroline adduct is a triaqua trihydrate (Scheme I) in which the 5-aminosophthlate dianion binds only through the neutral amino donor site; the coordinated water molecules comprise the *fac* points of the octahedron around the metal atom (Fig. 1). The zwitterionic dianion, the coordinated and lattice water molecules interact through hydrogen bonds (Table 2) to furnish a tightly-held, three-dimensional network. Pairs of phenanthroline units show π···π interactions about a center-of-inversion at a distance of *ca* 3.5 Å (Fig. 2).

### Experimental

Cobalt(II) nitrate hexahydrate (0.048 g, 0.165 mmol) dissolved in water (5 ml) was added to a mixture of 5-amino-isophthalic acid (0.030 g, 0.165 mmol) and sodium hydroxidie (0.013 g, 0.330 mmol) dissolved in water (5 ml). To this solution was added 1,10-phenanthroline (0.033 g, 0.165 mmol) dissolved in methanol (10 ml). The mixture was filtered and set aside for the growth of deep red crystals (35% yield based on the acid). CHN elemental analaysis. Calc. for C_20_H_25_C_O_N_3_O_10_: C 45.63, H 4.79, N 7.98%. Found: C, 45.49; H, 4.89; N,7.91%.

### Refinement

Hydrogen atoms were placed in calculated positions (C–H 0.93 Å) and were included in the refinement in the riding model approximation, with *U*_iso_(H) set to 1.2*U*_eq_(C). The amino and water bound H-atoms were located in difference Fourier maps, and were refined with a distance restraint of N–H = O–H = 0.85±0.01 Å. Their temperature factors were freely refined.

### Crystal data

**Table:**

[Co(C_8_H_5_NO_4_)(C_12_H_8_N_2_)(H_2_O)_3_]·3H_2_O	*F*(000) = 1092
*M**_r_* = 526.36	*D*_x_ = 1.527 Mg m^−^^3^
Monoclinic, *P*2_1_/*n*	Mo *K*α radiation, λ = 0.71073 Å
Hall symbol: -P 2yn	Cell parameters from 9790 reflections
*a* = 10.1182 (2) Å	θ = 2.5–28.5°
*b* = 13.9659 (2) Å	µ = 0.81 mm^−^^1^
*c* = 16.2850 (2) Å	*T* = 293 K
β = 95.827 (1)°	Prism, red
*V* = 2289.34 (8) Å^3^	0.24 × 0.22 × 0.18 mm
*Z* = 4	

### Data collection

**Table:**

Bruker APEXII area-detector diffractometer	5683 independent reflections
Radiation source: fine-focus sealed tube	5120 reflections with *I* > 2σ(*I*)
graphite	*R*_int_ = 0.027
φ and ω scans	θ_max_ = 28.5°, θ_min_ = 2.5°
Absorption correction: multi-scan (*SADABS*; Sheldrick, 1996)	*h* = −13→13
*T*_min_ = 0.803, *T*_max_ = 1.000	*k* = −18→18
18954 measured reflections	*l* = −21→21

### Refinement

**Table:**

Refinement on *F*^2^	Primary atom site location: structure-invariant direct methods
Least-squares matrix: full	Secondary atom site location: difference Fourier map
*R*[*F*^2^ > 2σ(*F*^2^)] = 0.028	Hydrogen site location: inferred from neighbouring sites
*wR*(*F*^2^) = 0.086	H atoms treated by a mixture of independent and constrained refinement
*S* = 1.04	*w* = 1/[σ^2^(*F*_o_^2^) + (0.0539*P*)^2^ + 0.5451*P*] where *P* = (*F*_o_^2^ + 2*F*_c_^2^)/3
5683 reflections	(Δ/σ)_max_ = 0.001
363 parameters	Δρ_max_ = 0.47 e Å^−^^3^
14 restraints	Δρ_min_ = −0.46 e Å^−^^3^

### Fractional atomic coordinates and isotropic or equivalent isotropic displacement parameters (Å^2^)

**Table:**

	*x*	*y*	*z*	*U*_iso_*/*U*_eq_	
Co1	0.742889 (15)	0.684399 (12)	0.466367 (10)	0.01480 (6)	
O1	1.15673 (10)	0.53092 (8)	0.26398 (8)	0.0313 (2)	
O2	1.22644 (9)	0.64316 (8)	0.18340 (7)	0.0283 (2)	
O3	0.92170 (9)	0.91482 (7)	0.09694 (6)	0.02231 (19)	
O4	0.72213 (9)	0.92307 (7)	0.14075 (6)	0.0237 (2)	
O1W	0.76586 (10)	0.70374 (8)	0.59335 (6)	0.0228 (2)	
H1W1	0.8296 (15)	0.6766 (13)	0.6202 (12)	0.036 (6)*	
H1W2	0.749 (2)	0.7538 (11)	0.6187 (13)	0.052 (7)*	
O2W	0.79629 (9)	0.54265 (7)	0.49718 (6)	0.01971 (18)	
H2W1	0.7468 (16)	0.5247 (15)	0.5321 (10)	0.037 (6)*	
H2W2	0.784 (2)	0.5056 (14)	0.4555 (10)	0.049 (6)*	
O3W	0.53714 (9)	0.66780 (8)	0.47286 (6)	0.0235 (2)	
H3W1	0.486 (2)	0.6450 (17)	0.4334 (11)	0.057 (7)*	
H3W2	0.509 (2)	0.6474 (15)	0.5168 (9)	0.042 (6)*	
O4W	0.44314 (12)	0.71848 (11)	0.29095 (9)	0.0424 (3)	
H4W1	0.451 (2)	0.7710 (11)	0.2661 (14)	0.056 (7)*	
H4W2	0.390 (2)	0.6844 (17)	0.2600 (15)	0.067 (9)*	
O5W	1.35918 (11)	0.52866 (8)	0.38701 (7)	0.0276 (2)	
H5W1	1.3070 (18)	0.5252 (16)	0.3431 (9)	0.041 (6)*	
H5W2	1.4231 (15)	0.4911 (13)	0.3801 (13)	0.040 (6)*	
O6W	1.02652 (9)	0.37775 (7)	0.31095 (7)	0.0248 (2)	
H6W1	1.063 (2)	0.4287 (10)	0.2960 (13)	0.044 (6)*	
H6W2	0.9517 (13)	0.3885 (15)	0.3285 (13)	0.045 (6)*	
N1	0.70534 (10)	0.63853 (8)	0.33691 (6)	0.0168 (2)	
H1N1	0.6259 (11)	0.6547 (13)	0.3202 (11)	0.023 (4)*	
H1N2	0.7054 (19)	0.5782 (7)	0.3384 (12)	0.031 (5)*	
N2	0.71726 (11)	0.83333 (8)	0.43935 (7)	0.0198 (2)	
N3	0.94173 (10)	0.73017 (8)	0.45306 (7)	0.0186 (2)	
C1	0.79644 (12)	0.67271 (9)	0.28227 (7)	0.0155 (2)	
C2	0.91651 (12)	0.62476 (9)	0.27734 (7)	0.0166 (2)	
H2	0.9344	0.5688	0.3074	0.020*	
C3	1.00960 (11)	0.66020 (9)	0.22771 (7)	0.0160 (2)	
C4	0.98255 (12)	0.74438 (9)	0.18275 (8)	0.0170 (2)	
H4	1.0446	0.7686	0.1498	0.020*	
C5	0.86219 (11)	0.79214 (9)	0.18732 (7)	0.0158 (2)	
C6	0.76966 (12)	0.75642 (9)	0.23778 (7)	0.0168 (2)	
H6	0.6902	0.7888	0.2415	0.020*	
C7	1.14016 (11)	0.60753 (9)	0.22423 (8)	0.0180 (2)	
C8	0.83353 (12)	0.88295 (9)	0.13858 (7)	0.0164 (2)	
C9	0.60574 (14)	0.88414 (11)	0.43592 (9)	0.0264 (3)	
H9	0.5278	0.8534	0.4466	0.032*	
C10	0.60072 (16)	0.98217 (12)	0.41679 (10)	0.0316 (3)	
H10	0.5210	1.0156	0.4157	0.038*	
C11	0.71446 (17)	1.02817 (11)	0.39974 (9)	0.0307 (3)	
H11	0.7127	1.0932	0.3875	0.037*	
C12	0.83406 (15)	0.97630 (10)	0.40083 (9)	0.0259 (3)	
C13	0.83020 (13)	0.87881 (10)	0.42259 (8)	0.0195 (2)	
C14	0.95735 (17)	1.01683 (12)	0.38069 (11)	0.0365 (4)	
H14	0.9602	1.0810	0.3657	0.044*	
C15	1.06906 (17)	0.96363 (13)	0.38306 (12)	0.0379 (4)	
H15	1.1469	0.9912	0.3682	0.045*	
C16	1.06940 (14)	0.86486 (12)	0.40818 (10)	0.0280 (3)	
C17	0.95058 (13)	0.82265 (10)	0.42820 (8)	0.0198 (2)	
C18	1.18338 (15)	0.80580 (13)	0.41512 (11)	0.0353 (4)	
H18	1.2640	0.8297	0.4013	0.042*	
C19	1.17504 (14)	0.71351 (13)	0.44210 (11)	0.0326 (3)	
H19	1.2503	0.6749	0.4482	0.039*	
C20	1.05169 (13)	0.67760 (10)	0.46052 (9)	0.0245 (3)	
H20	1.0469	0.6147	0.4786	0.029*	

### Atomic displacement parameters (Å^2^)

**Table:**

	*U*^11^	*U*^22^	*U*^33^	*U*^12^	*U*^13^	*U*^23^
Co1	0.01501 (9)	0.01376 (10)	0.01610 (10)	0.00058 (5)	0.00396 (6)	0.00020 (6)
O1	0.0198 (4)	0.0260 (5)	0.0490 (7)	0.0059 (4)	0.0081 (4)	0.0196 (5)
O2	0.0216 (4)	0.0249 (5)	0.0407 (6)	0.0061 (4)	0.0137 (4)	0.0139 (5)
O3	0.0215 (4)	0.0204 (5)	0.0265 (5)	0.0018 (4)	0.0095 (3)	0.0082 (4)
O4	0.0216 (4)	0.0192 (5)	0.0320 (5)	0.0066 (4)	0.0109 (4)	0.0087 (4)
O1W	0.0260 (5)	0.0237 (5)	0.0185 (4)	0.0059 (4)	0.0009 (4)	−0.0061 (4)
O2W	0.0266 (4)	0.0146 (4)	0.0182 (4)	−0.0006 (4)	0.0031 (3)	−0.0010 (4)
O3W	0.0170 (4)	0.0344 (5)	0.0198 (5)	−0.0033 (4)	0.0045 (3)	−0.0009 (4)
O4W	0.0265 (5)	0.0461 (8)	0.0526 (8)	−0.0063 (5)	−0.0051 (5)	0.0224 (7)
O5W	0.0306 (5)	0.0256 (5)	0.0269 (5)	0.0060 (4)	0.0045 (4)	0.0030 (4)
O6W	0.0206 (4)	0.0192 (5)	0.0354 (5)	0.0003 (4)	0.0073 (4)	0.0018 (4)
N1	0.0181 (4)	0.0166 (5)	0.0165 (5)	−0.0001 (4)	0.0058 (4)	0.0032 (4)
N2	0.0202 (5)	0.0180 (5)	0.0214 (5)	0.0022 (4)	0.0036 (4)	0.0007 (4)
N3	0.0171 (5)	0.0184 (5)	0.0205 (5)	−0.0001 (4)	0.0025 (4)	−0.0015 (4)
C1	0.0175 (5)	0.0165 (5)	0.0132 (5)	−0.0008 (4)	0.0041 (4)	0.0009 (4)
C2	0.0192 (5)	0.0147 (5)	0.0161 (5)	0.0009 (4)	0.0024 (4)	0.0027 (4)
C3	0.0157 (5)	0.0152 (5)	0.0172 (5)	0.0011 (4)	0.0022 (4)	0.0005 (4)
C4	0.0171 (5)	0.0167 (6)	0.0180 (5)	0.0000 (4)	0.0051 (4)	0.0024 (5)
C5	0.0175 (5)	0.0139 (5)	0.0166 (5)	0.0008 (4)	0.0041 (4)	0.0022 (4)
C6	0.0168 (5)	0.0172 (6)	0.0171 (5)	0.0020 (4)	0.0051 (4)	0.0006 (5)
C7	0.0152 (5)	0.0166 (6)	0.0222 (6)	0.0011 (4)	0.0020 (4)	0.0028 (5)
C8	0.0194 (5)	0.0136 (5)	0.0164 (5)	0.0008 (4)	0.0035 (4)	0.0018 (4)
C9	0.0251 (6)	0.0243 (7)	0.0308 (7)	0.0063 (5)	0.0066 (5)	0.0030 (6)
C10	0.0378 (8)	0.0258 (7)	0.0313 (7)	0.0149 (6)	0.0044 (6)	0.0046 (6)
C11	0.0465 (8)	0.0180 (6)	0.0267 (7)	0.0059 (6)	−0.0005 (6)	0.0034 (6)
C12	0.0349 (7)	0.0185 (6)	0.0236 (6)	−0.0026 (5)	−0.0004 (5)	0.0037 (5)
C13	0.0234 (6)	0.0172 (6)	0.0178 (5)	−0.0011 (5)	0.0019 (4)	0.0006 (5)
C14	0.0441 (9)	0.0232 (7)	0.0416 (9)	−0.0111 (6)	0.0012 (7)	0.0102 (7)
C15	0.0344 (8)	0.0343 (9)	0.0454 (9)	−0.0156 (7)	0.0063 (7)	0.0089 (7)
C16	0.0239 (6)	0.0296 (7)	0.0309 (7)	−0.0085 (6)	0.0050 (5)	0.0024 (6)
C17	0.0196 (6)	0.0199 (6)	0.0201 (6)	−0.0025 (5)	0.0026 (4)	−0.0012 (5)
C18	0.0186 (6)	0.0439 (10)	0.0441 (9)	−0.0075 (6)	0.0068 (6)	−0.0006 (7)
C19	0.0166 (6)	0.0376 (8)	0.0436 (9)	0.0031 (6)	0.0034 (6)	−0.0033 (7)
C20	0.0202 (6)	0.0229 (7)	0.0302 (7)	0.0027 (5)	0.0011 (5)	−0.0025 (5)

### Geometric parameters (Å, °)

**Table:**

Co1—O1W	2.0750 (10)	C1—C2	1.3967 (16)
Co1—O2W	2.0995 (9)	C2—C3	1.3927 (16)
Co1—O3W	2.1080 (9)	C2—H2	0.9300
Co1—N2	2.1364 (12)	C3—C4	1.3974 (17)
Co1—N3	2.1429 (10)	C3—C7	1.5181 (16)
Co1—N1	2.1992 (11)	C4—C5	1.3971 (16)
O1—C7	1.2530 (16)	C4—H4	0.9300
O2—C7	1.2530 (15)	C5—C6	1.3989 (16)
O3—C8	1.2556 (14)	C5—C8	1.5086 (17)
O4—C8	1.2624 (15)	C6—H6	0.9300
O1W—H1W1	0.83 (1)	C9—C10	1.404 (2)
O1W—H1W2	0.84 (1)	C9—H9	0.9300
O2W—H2W1	0.83 (1)	C10—C11	1.371 (2)
O2W—H2W2	0.85 (1)	C10—H10	0.9300
O3W—H3W1	0.84 (1)	C11—C12	1.409 (2)
O3W—H3W2	0.85 (1)	C11—H11	0.9300
O4W—H4W1	0.85 (1)	C12—C13	1.4085 (19)
O4W—H4W2	0.85 (1)	C12—C14	1.438 (2)
O5W—H5W1	0.85 (1)	C13—C17	1.4437 (18)
O5W—H5W2	0.85 (1)	C14—C15	1.350 (3)
O6W—H6W1	0.85 (1)	C14—H14	0.9300
O6W—H6W2	0.85 (1)	C15—C16	1.439 (2)
N1—C1	1.4269 (15)	C15—H15	0.9300
N1—H1N1	0.85 (1)	C16—C17	1.4066 (18)
N1—H1N2	0.84 (1)	C16—C18	1.413 (2)
N2—C9	1.3293 (17)	C18—C19	1.367 (2)
N2—C13	1.3590 (17)	C18—H18	0.9300
N3—C20	1.3282 (17)	C19—C20	1.405 (2)
N3—C17	1.3593 (17)	C19—H19	0.9300
C1—C6	1.3876 (17)	C20—H20	0.9300
			
O1W—Co1—O2W	83.37 (4)	C3—C4—H4	120.0
O1W—Co1—O3W	88.60 (4)	C4—C5—C6	120.00 (11)
O2W—Co1—O3W	96.65 (4)	C4—C5—C8	119.70 (10)
O1W—Co1—N2	94.45 (4)	C6—C5—C8	120.29 (11)
O2W—Co1—N2	171.97 (4)	C1—C6—C5	120.02 (11)
O3W—Co1—N2	91.01 (4)	C1—C6—H6	120.0
O1W—Co1—N3	92.90 (4)	C5—C6—H6	120.0
O2W—Co1—N3	94.82 (4)	O2—C7—O1	123.33 (11)
O3W—Co1—N3	168.53 (4)	O2—C7—C3	118.90 (11)
N2—Co1—N3	77.54 (4)	O1—C7—C3	117.76 (11)
O1W—Co1—N1	169.81 (4)	O3—C8—O4	122.90 (11)
O2W—Co1—N1	88.26 (4)	O3—C8—C5	118.22 (11)
O3W—Co1—N1	86.60 (4)	O4—C8—C5	118.88 (10)
N2—Co1—N1	94.62 (4)	N2—C9—C10	122.77 (14)
N3—Co1—N1	93.59 (4)	N2—C9—H9	118.6
Co1—O1W—H1W1	117.8 (15)	C10—C9—H9	118.6
Co1—O1W—H1W2	126.2 (16)	C11—C10—C9	119.33 (14)
H1W1—O1W—H1W2	108 (2)	C11—C10—H10	120.3
Co1—O2W—H2W1	106.8 (15)	C9—C10—H10	120.3
Co1—O2W—H2W2	111.5 (16)	C10—C11—C12	119.61 (14)
H2W1—O2W—H2W2	108 (2)	C10—C11—H11	120.2
Co1—O3W—H3W1	122.1 (17)	C12—C11—H11	120.2
Co1—O3W—H3W2	120.1 (15)	C13—C12—C11	117.03 (13)
H3W1—O3W—H3W2	106 (2)	C13—C12—C14	119.00 (14)
H4W1—O4W—H4W2	107 (2)	C11—C12—C14	123.96 (14)
H5W1—O5W—H5W2	106 (2)	N2—C13—C12	123.20 (12)
H6W1—O6W—H6W2	112 (2)	N2—C13—C17	117.04 (12)
C1—N1—Co1	116.15 (8)	C12—C13—C17	119.75 (12)
C1—N1—H1N1	111.1 (12)	C15—C14—C12	121.35 (14)
Co1—N1—H1N1	107.1 (12)	C15—C14—H14	119.3
C1—N1—H1N2	110.7 (13)	C12—C14—H14	119.3
Co1—N1—H1N2	105.3 (13)	C14—C15—C16	120.90 (14)
H1N1—N1—H1N2	105.8 (18)	C14—C15—H15	119.5
C9—N2—C13	118.01 (12)	C16—C15—H15	119.5
C9—N2—Co1	127.81 (10)	C17—C16—C18	116.54 (14)
C13—N2—Co1	114.17 (9)	C17—C16—C15	119.36 (14)
C20—N3—C17	118.20 (11)	C18—C16—C15	124.11 (14)
C20—N3—Co1	127.75 (10)	N3—C17—C16	123.43 (13)
C17—N3—Co1	113.86 (8)	N3—C17—C13	117.02 (11)
C6—C1—C2	119.92 (11)	C16—C17—C13	119.55 (13)
C6—C1—N1	120.22 (11)	C19—C18—C16	119.97 (14)
C2—C1—N1	119.75 (11)	C19—C18—H18	120.0
C3—C2—C1	120.43 (11)	C16—C18—H18	120.0
C3—C2—H2	119.8	C18—C19—C20	119.35 (14)
C1—C2—H2	119.8	C18—C19—H19	120.3
C2—C3—C4	119.67 (11)	C20—C19—H19	120.3
C2—C3—C7	119.46 (11)	N3—C20—C19	122.48 (14)
C4—C3—C7	120.86 (11)	N3—C20—H20	118.8
C5—C4—C3	119.95 (11)	C19—C20—H20	118.8
C5—C4—H4	120.0		
			
O1W—Co1—N1—C1	−145.0 (2)	C4—C5—C8—O3	−2.25 (18)
O2W—Co1—N1—C1	−110.28 (9)	C6—C5—C8—O3	176.88 (12)
O3W—Co1—N1—C1	152.95 (9)	C4—C5—C8—O4	177.14 (12)
N2—Co1—N1—C1	62.21 (9)	C6—C5—C8—O4	−3.73 (18)
N3—Co1—N1—C1	−15.56 (9)	C13—N2—C9—C10	−0.8 (2)
O1W—Co1—N2—C9	−85.16 (12)	Co1—N2—C9—C10	−179.49 (11)
O3W—Co1—N2—C9	3.51 (12)	N2—C9—C10—C11	0.9 (2)
N3—Co1—N2—C9	−177.16 (13)	C9—C10—C11—C12	0.7 (2)
N1—Co1—N2—C9	90.18 (12)	C10—C11—C12—C13	−2.1 (2)
O1W—Co1—N2—C13	96.06 (9)	C10—C11—C12—C14	177.43 (16)
O3W—Co1—N2—C13	−175.26 (9)	C9—N2—C13—C12	−0.9 (2)
N3—Co1—N2—C13	4.07 (9)	Co1—N2—C13—C12	178.02 (10)
N1—Co1—N2—C13	−88.59 (9)	C9—N2—C13—C17	178.87 (12)
O1W—Co1—N3—C20	85.77 (12)	Co1—N2—C13—C17	−2.23 (15)
O2W—Co1—N3—C20	2.19 (12)	C11—C12—C13—N2	2.3 (2)
O3W—Co1—N3—C20	−176.94 (17)	C14—C12—C13—N2	−177.27 (14)
N2—Co1—N3—C20	179.69 (12)	C11—C12—C13—C17	−177.44 (13)
N1—Co1—N3—C20	−86.36 (12)	C14—C12—C13—C17	3.0 (2)
O1W—Co1—N3—C17	−99.37 (9)	C13—C12—C14—C15	−0.7 (2)
O2W—Co1—N3—C17	177.06 (9)	C11—C12—C14—C15	179.79 (16)
O3W—Co1—N3—C17	−2.1 (3)	C12—C14—C15—C16	−1.8 (3)
N2—Co1—N3—C17	−5.45 (9)	C14—C15—C16—C17	1.9 (3)
N1—Co1—N3—C17	88.50 (9)	C14—C15—C16—C18	−177.55 (17)
Co1—N1—C1—C6	−92.87 (12)	C20—N3—C17—C16	1.5 (2)
Co1—N1—C1—C2	83.33 (13)	Co1—N3—C17—C16	−173.89 (11)
C6—C1—C2—C3	−0.27 (19)	C20—N3—C17—C13	−178.51 (12)
N1—C1—C2—C3	−176.48 (11)	Co1—N3—C17—C13	6.10 (15)
C1—C2—C3—C4	0.13 (19)	C18—C16—C17—N3	−0.1 (2)
C1—C2—C3—C7	179.20 (11)	C15—C16—C17—N3	−179.52 (14)
C2—C3—C4—C5	−0.45 (19)	C18—C16—C17—C13	179.94 (14)
C7—C3—C4—C5	−179.51 (11)	C15—C16—C17—C13	0.5 (2)
C3—C4—C5—C6	0.91 (19)	N2—C13—C17—N3	−2.64 (18)
C3—C4—C5—C8	−179.95 (11)	C12—C13—C17—N3	177.12 (12)
C2—C1—C6—C5	0.73 (19)	N2—C13—C17—C16	177.35 (12)
N1—C1—C6—C5	176.92 (11)	C12—C13—C17—C16	−2.9 (2)
C4—C5—C6—C1	−1.06 (19)	C17—C16—C18—C19	−1.6 (2)
C8—C5—C6—C1	179.81 (11)	C15—C16—C18—C19	177.86 (17)
C2—C3—C7—O2	−175.69 (12)	C16—C18—C19—C20	1.7 (3)
C4—C3—C7—O2	3.38 (19)	C17—N3—C20—C19	−1.3 (2)
C2—C3—C7—O1	3.05 (18)	Co1—N3—C20—C19	173.33 (11)
C4—C3—C7—O1	−177.88 (13)	C18—C19—C20—N3	−0.3 (2)

### Hydrogen-bond geometry (Å, °)

**Table:**

*D*—H···*A*	*D*—H	H···*A*	*D*···*A*	*D*—H···*A*
O1w—H1w1···O6w^i^	0.83 (1)	1.90 (1)	2.734 (1)	175 (2)
O1w—H1w2···O2^ii^	0.84 (1)	1.81 (1)	2.646 (1)	173 (2)
O2w—H2w1···O5w^i^	0.83 (1)	1.93 (1)	2.761 (1)	174 (2)
O2w—H2w2···O4^iii^	0.85 (1)	1.94 (1)	2.790 (1)	173 (2)
O3w—H3w1···O5w^iv^	0.84 (1)	2.16 (2)	2.909 (2)	148 (2)
O3w—H3w2···O3^ii^	0.85 (1)	1.86 (1)	2.694 (1)	167 (2)
O4w—H4w1···O6w^v^	0.85 (1)	1.98 (1)	2.811 (2)	169 (2)
O4w—H4w2···O2^iv^	0.85 (1)	2.05 (1)	2.864 (2)	161 (3)
O5w—H5w1···O1	0.85 (1)	1.89 (1)	2.716 (2)	164 (2)
O5w—H5w2···O3^vi^	0.85 (1)	1.90 (1)	2.719 (1)	161 (2)
O6w—H6w1···O1	0.85 (1)	1.82 (1)	2.665 (1)	174 (2)
O6w—H6w2···O4^iii^	0.85 (1)	1.94 (1)	2.784 (1)	174 (2)
N1—H1N1···O4w	0.85 (1)	2.06 (1)	2.906 (2)	169 (2)
N1—H1N2···O4^iii^	0.84 (1)	2.30 (1)	3.110 (2)	161 (2)

Symmetry codes: (i) −*x*+2, −*y*+1, −*z*+1; (ii) *x*−1/2, −*y*+3/2, *z*+1/2; (iii) −*x*+3/2, *y*−1/2, −*z*+1/2; (iv) *x*−1, *y*, *z*; (v) −*x*+3/2, *y*+1/2, −*z*+1/2; (vi) −*x*+5/2, *y*−1/2, −*z*+1/2.
